# Erythroplasia of Queyrat treated with imiquimod 5% cream: The necessity of regimen guidelines

**DOI:** 10.1002/ccr3.2076

**Published:** 2019-02-27

**Authors:** Maiko Yokoyama, Gyohei Egawa, Takamitsu Makino, Kiyofumi Egawa

**Affiliations:** ^1^ Division of Dermatology Amakusa Hosipital Amakusa Japan; ^2^ Department of Dermatology, Graduate School of Medicine Kyoto University Kyoto Japan; ^3^ Department of Dermatology and Plastic Surgery, Faculty of Life Sciences Kumamoto University Kumamoto Japan; ^4^ Division of Dermatology, Amakusa Dermatology and Internal Medicine Clinic Kamiamakusa Japan; ^5^ Department of Dermatology Kumamoto University Hospital Kumamoto Japan

**Keywords:** carcinoma in situ, erythroplasia of Queyrat, imiquimod, regimen guideline

## Abstract

Development of noninvasive treatments for erythroplasia of Queyrat, a carcinoma in situ, is expected. This case suggests topical imiquimod might be a candidate with regimens consisting of much longer duration of the treatment than for genital warts and the maintenance phase of the treatment course to prevent the relapse.

## 1 INTRODUCTION

Erythroplasia of Queyrat (EQ) is a carcinoma in situ that appears as a well‐marginated erythematous velvety patch or plaque mainly on the glans penis.^1^ While EQ has classically required surgical treatment because of the risk of progression to invasive carcinoma, the patients often suffer from poor cosmetic and functional outcomes.^2^ Although there are several reports evaluating the efficacy of topical imiquimod (IQ) as an alternative noninvasive treatment for EQ,^3^ there is currently no standard regimen treating EQ with IQ, owing to the small series of reported cases.

## 2 CASE REPORT

**Figure 1:**
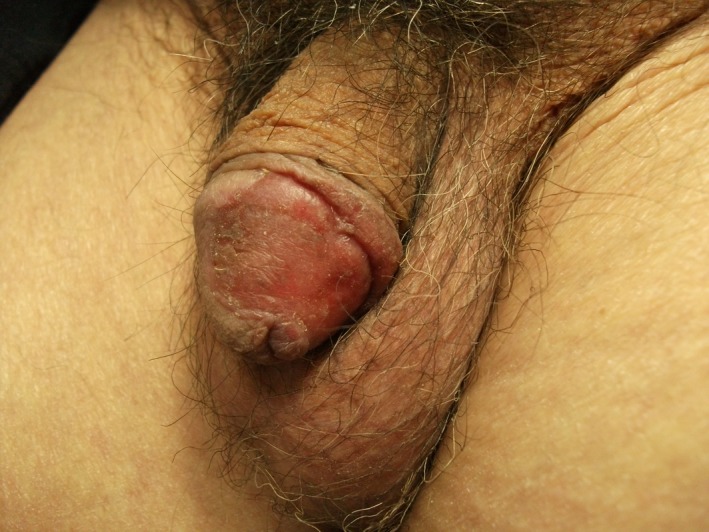
Clinical presentation before the treatment. EQ of the glans and foreskin of the penis, appearing as a shiny, erythematous, and slightly infiltrated plaque

**Figure 2:**
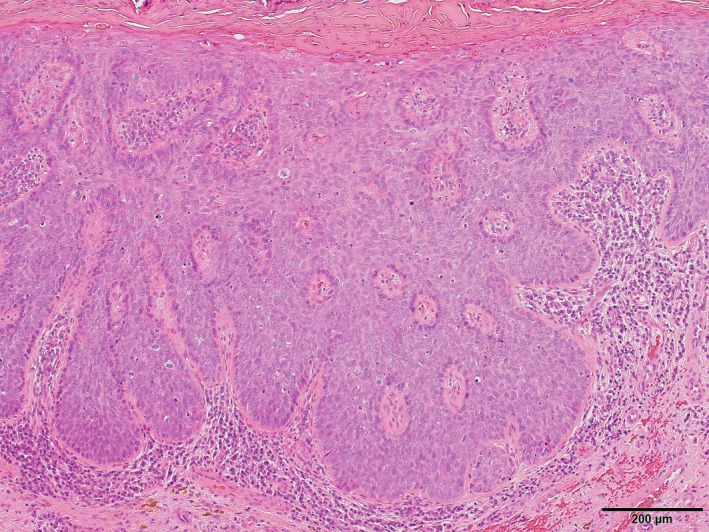
Histopathology examination showed irregularly acanthotic squamous epithelium with markedly atypical keratinocytes, many mitoses, and a loss of polarity but without invasion, thus confirming the diagnosis of a carcinoma in situ (H&E stain, original magnification ×100)

A 77‐year‐old male suffering from Alzheimer's disease presented with a persistent erythematous lesion of several years’ duration on the glans penis. On physical examination, an asymptomatic, sharply demarcated, erythematous, partially erosive plaque was observed on the glans penis (Figure 1). A penile biopsy showed a carcinoma in situ (EQ)^1^ (Figure 2). Polymerase chain reaction (PCR)^4^ analysis with DNA extract of the skin biopsy specimen demonstrated human papillomavirus (HPV16) DNA.^5^ The patient showed no clinical or laboratory signs of immunodeficiency; no metastases were detected by chest and abdominal computed tomography (CT) scan.

**Figure 3:**
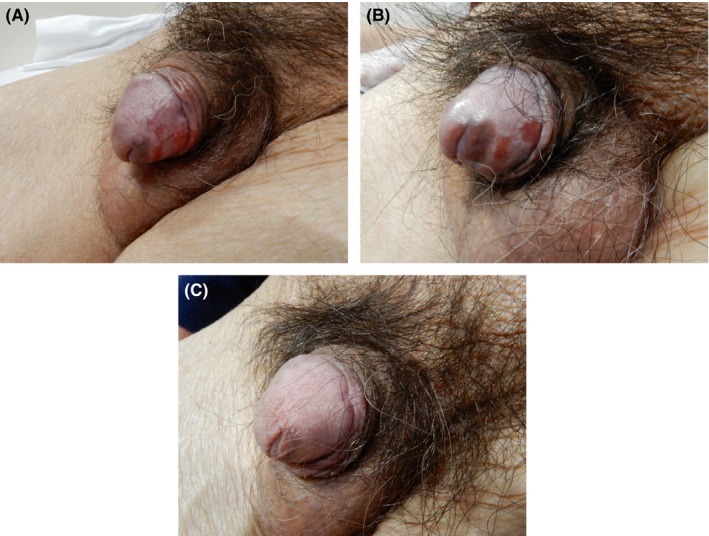
A, Partial resolution of the lesion after 16 wk of imiquimod treatment. B, Before the 2nd course of the treatment: smaller erosive lesions on the glans penis. C, The final clinical resolution after 5 wk of the 3rd course of the treatment

The whole glans was treated three times a week for 16 weeks with IQ 5% cream in accordance with a standard regimen for genital warts.^6^ With the treatment, the lesion was substantially decreased but small erosion was remained (Figure 3A). The erosive lesion gradually became smaller, but not completely disappeared (Figure 3B). Therefore, after a 7‐week interval, we again applied IQ for 12 weeks and the lesion was clinically disappeared. Five weeks later, however, small erosion was relapsed and another course of IQ application was performed for 5 weeks until the erosion was disappeared (Figure 3C).

During the courses of the treatment, the patient experienced a moderate burning sensation at the applied area, otherwise he tolerated the procedure well. The patient is now carefully followed up to survey the relapse. To date, 21 weeks after completion of the imiquimod therapy, patient is relapse free.

## 3 DISCUSSION

Imiquimod is a topical immune response modifier that activates macrophages and other cells to stimulate both innate and adaptive immune systems, mainly via Toll‐like receptor 7 agonism. Ultimately, this leads to immune defense against both viral‐infected cells and tumor cells and thus is effective for the treatment of HPV‐associated skin disorders and nonmelanoma skin cancers.^7^, ^8^ IQ is initially approved by the US Food and Drug Administration (FDA) for the treatment of external genital or perianal warts, and later for face and scalp actinic keratoses (AKs) and superficial basal cell carcinomas (BCCs).^7^, ^8^, ^9^, ^10^

Our observation is similar to the reported outcomes in which IQ was suggested to be a therapeutic option in EQ,^2^, ^3^ especially in individuals who cannot bear surgical operation. However, in the present case, EQ not only took much longer total duration of the IQ application to achieve final clinical resolution than genital warts but also relapsed soon after the first clinical resolution.

Recent literatures suggest that an initial treatment regimen with a longer total duration of topical IQ may be more effective for EQ^3^ and that the maintenance phase of the treatment course might be necessary to prevent the relapse.^2^

Taken together, we suppose that a specific regimen must be established for the treatment of EQ with IQ. The regimen we would propose may consist of much longer duration of the treatment than that for genital warts and a maintenance phase of the treatment course to prevent the relapse. Furthermore, combined therapy with cryotherapy,^2^ topical 5‐FU,^8^, ^10^ or laser therapy^11^ may be alternative treatment for EQ which shows partial response to such an IQ monotherapy.

## CONFLICT OF INTEREST

None declared.

## AUTHOR CONTRIBUTION

MY: provided medical care, made the diagnosis, treated the patient, and wrote the first draft of the report. GE: conducted PCR analysis with DNA extract of the skin biopsy specimen. TM: conducted a penile skin biopsy and histological evaluation of the lesion. KE: conducted a critical review of the literature, critically revised the manuscript, and supervised the study. All authors read and approved the final version of the manuscript.
